# Intraspecific Physiological Alterations in Phytoplankton Declined the Food Nutritional Quality in Tropical Nutrient-Manipulated Aquatic Mesocosms

**DOI:** 10.3390/microorganisms14092060

**Published:** 2026-09-15

**Authors:** Weiran Feng, Qiuqi Lin, Shuping Liang, Mingjie Li, Vladimir Razlutskij, Zhengwen Liu, Jian Gao, Yali Tang

**Affiliations:** 1 Department of Ecology, Jinan University, Guangzhou 510632, China; fengwr10@163.com (W.F.); qqlin@jnu.edu.cn (Q.L.); l2979396392@163.com (S.L.); limj09@stu2025.jnu.edu.cn (M.L.); 2 Engineering Research Center of Tropical and Subtropical Aquatic Ecological Engineering, Ministry of Education, Jinan University, 601 Huangpu Avenue West, Guangzhou 510632, China; 3 State Scientific and Production Amalgamation Scientific-Practical Center of the National Academy of Sciences of Belarus for Biological Resources, 220072 Minsk, Belarus; vladimirrazl@mail.ru; 4 State Key Laboratory of Lake Science and Environment, Institute of Geography and Limnology, Chinese Academy of Sciences, Nanjing 210008, China; zliu@niglas.ac.cn; 5 Key Laboratory of Intelligent Health Perception and Ecological Restoration of Rivers and Lakes, Ministry of Education, Hubei University of Technology, Wuhan 430070, China

**Keywords:** ω-3 PUFA, phytoplankton community diversity, food nutritional quality, phosphorus, eutrophic aquatic ecosystems

## Abstract

The polyunsaturated fatty acid (PUFA) content of phytoplankton determines the nutritional quality of aquatic basal food resources and influences food-web energy transfer. Field studies suggest that phytoplankton PUFA content is associated with taxonomic characteristics, with nutrient enrichment promoting cyanobacteria dominance to reduce phytoplankton PUFA content. Nevertheless, laboratory evidence indicates that nutrient enrichment can also alter the fatty acid composition of single algae species. The role of intraspecific physiological alterations has long been overlooked. To partition the relative importance of taxonomic and intraspecific physiological alterations on phytoplankton nutritional quality, nutrient-manipulated mesocosm experiments were conducted in spring and winter using natural plankton communities from a tropical eutrophic reservoir. Fatty acid analysis showed that the nutritional quality of the phytoplankton community declined in mesocosms after nutrient additions. Piecewise structural equation modeling (pSEM) was employed to disentangle direct effects (representing intraspecific physiological alterations) and indirect effects (via the Shannon–Wiener index, representing taxonomic alterations) of phosphorus enrichment on ω-3 PUFA. Within each season, phosphorus enrichment exerted a significant direct negative effect on ω-3 PUFA, regardless of whether cyanobacteria dominated in winter or chlorophytes dominated in spring. In the pooled cross-seasonal model, community diversity showed a significant positive effect on ω-3 PUFA, with a stronger standardized effect than that of phosphorus enrichment, whereas phosphorus enrichment still exerted a significant direct negative effect. Our results indicate that intraspecific physiological alterations in phytoplankton may play a crucial role in the changes in phytoplankton nutritional quality following additional nutrient loading, particularly in tropical, eutrophic waters. However, the underlying biochemical mechanisms remain uncertain because direct molecular or enzymatic evidence was not obtained in this study.

## 1. Introduction

Food quality stands as one of the crucial factors in sustaining the stability of freshwater food webs and exerting an influence on aquaculture [1,2,3]. Food quality encompasses a multitude of nutritional constituents, including elemental composition, protein content, and fatty acid composition. In particular, omega-3 polyunsaturated fatty acids (ω-3 PUFA) are essential nutrients for both aquatic and terrestrial organisms [4,5,6]. They are indispensable structural elements of cell membranes, play a role in the regulation of transmembrane transport, and act as precursors of key hormones involved in embryonic development and immune regulation [7,8,9]. A dietary deficiency of PUFA can impede the normal growth and reproduction of aquatic consumers, such as zooplankton and fish [10,11,12,13], ultimately impacting the energy transfer within the food web and aquaculture [3,14,15,16]. Therefore, ω-3 PUFA is widely recognized as a key indicator of food nutritional quality.

Phytoplankton within aquatic ecosystems serve as the primary origin of ω-3 PUFA in the biosphere [17,18]. The ω-3 PUFA content of phytoplankton exhibits variations across taxonomic groups. Cryptophytes, dinoflagellates, and diatoms possess high levels of long-chain ω-3 PUFAs, such as eicosapentaenoic acid (EPA) and docosahexaenoic acid (DHA), and are thus regarded as high-quality food sources. Chlorophytes are characterized by high concentrations of C18 ω-3 PUFA, particularly α-linolenic acid (ALA), which can serve as a precursor for the biosynthesis of long-chain ω-3 PUFA in some aquatic consumers. Therefore, chlorophytes are generally considered food sources of intermediate nutritional quality. Conversely, cyanobacteria are devoid of long-chain ω-3 PUFAs and are generally regarded as poor-quality food sources [1,19,20,21,22,23]. Consequently, alterations in algal community composition can modify PUFA concentrations in seston [24], thereby impacting the nutritional quality of basal food resources and ultimately affecting energy transfer processes within aquatic food chains and aquaculture.

Nitrogen and phosphorus are among the major determinants of phytoplankton community structure. They serve as the primary limiting nutrients for phytoplankton growth [25,26,27]. Phytoplankton species exhibit significant disparities in functional traits, including nutrient uptake rates, half-saturation constants, maximum growth rates, and cell size. These disparities result in substantial variations in nutrient requirements and ultimately determine their competitive advantages under different nitrogen and phosphorus conditions [28,29,30]. Generally, excessive nutrient enrichment may enhance the relative abundance of low-quality algae while diminishing that of high-quality taxa, leading to an overall decrease in PUFA concentrations [31,32,33]. It is widely acknowledged that elevated phosphorus levels are correlated with significant reductions in EPA and DHA, whereas nitrogen enrichment typically leads to decreased ω-3 PUFA concentrations [32,33,34,35,36].

Nitrogen and phosphorus concentrations not only modify the taxonomic groups within phytoplankton but may also alter their PUFA content by inducing intraspecific physiological changes [37,38,39]. Nutrient availability serves as a crucial factor in regulating fatty acid synthesis and composition in algae. For instance, nitrogen limitation reduces fatty acid (FA) synthesis in Chlorophyta *Chlorella sorokiniana* and Chlorophyta *Chlamydomonas reinhardtii* [40], yet nitrogen deprivation increases FA accumulation in Haptophyta *Isochrysis galbana* [41]. Nitrogen availability can also modulate FA composition by influencing the activity of fatty acid desaturases. When nitrogen is sufficient and *Isochrysis* aff. *galbana* is in the exponential growth phase, the expression of fatty acid desaturases, such as d5FAD and d4FAD, is significantly upregulated, thereby promoting PUFA synthesis [42].

Phosphorus limitation results in a higher total FA concentration but a lower proportion of PUFA in Chlorophyta *Chlamydomonas acidophila* [43]. High phosphorus levels lead to a decrease in the FA and unsaturated fatty acid content of Rhodophyta *Porphyridium purpureum* [39]. Under phosphorus-limited conditions, algae can adapt to phosphorus deficiency by degrading phospholipids and synthesizing phosphorus-free lipids, which may lead to changes in fatty acid composition [44,45]. Phosphorus concentration can also regulate the transcription or expression of algal desaturases and elongases to influence the content of ω-3 PUFA [46,47,48,49].

In addition, under nitrogen or phosphorus deficiency, the antioxidant enzyme activity of *Chlorella pyrenoidosa* increases significantly, which induces lipid accumulation and alters fatty acid composition, characterized by an increased proportion of saturated fatty acids (SAFA). With increasing nutrient supply, the FA composition shifts towards a higher proportion of PUFA [50]. Furthermore, changes in nutrient stoichiometry may also influence fatty acid composition. At higher N:P ratios, algae tend to exhibit higher proportions of SAFA and monounsaturated fatty acids (MUFA), whereas the proportion of PUFA generally declines [38].

Overall, nitrogen and phosphorus availability can modify algal fatty acid composition by regulating fatty acid synthesis and desaturation processes, although the responses to nutrient conditions vary substantially among algal species. At present, limnological understanding is largely based on the perspective that variations in phytoplankton fatty acid composition primarily stem from taxonomic differences among algal groups. In contrast, studies addressing fatty acid changes driven by algal physiological responses are mostly derived from single-species culture experiments. In freshwater ecosystems, where multiple algal taxa coexist, the direct physiological effects of nutrient changes on community-level fatty acid composition remain poorly understood, and relevant empirical evidence is still limited.

Against the backdrop of widespread global lake eutrophication, it is hypothesized that the nutrient-enrichment-induced decline in the nutritional quality of aquatic basal food resources is not solely attributable to the indirect effects mediated by changes in phytoplankton community structure but may also potentially result from direct intraspecific physiological effects on phytoplankton. Based on this hypothesis, a mesocosm experiment was carried out using reservoir water without microplankton filtration from the Dashahe Reservoir in Kaiping City, Jiangmen, Guangdong Province. By means of controlled additions of nitrogen and phosphorus, the impacts of nitrogen and/or phosphorus enrichment on phytoplankton biomass, community structure, and diversity, as well as on their fatty acid profiles in the water, were investigated. Piecewise structural equation modeling (pSEM) was employed to distinguish the direct and indirect effects of nutrient enrichment on the nutritional quality of phytoplankton. The findings offer mechanistic understandings of the changes in basal food quality within aquatic food webs and enhance our comprehension of how eutrophication drives alterations in aquatic food web structure and the underlying mechanisms.

## 2. Materials and Methods

### 2.1. Experimental Design

To validate our hypotheses, winter (from 17 November 2022 to 5 January 2023) and spring (from 27 April to 6 July 2023) experiments were carried out at the Dashahe Reservoir in Kaiping City, Jiangmen, Guangdong Province, China. The experiment utilized twelve polyethylene mesocosms (with a diameter of 123 cm, a height of 71 cm, and an approximate volume of 1000 L). A 3 W wave-generating pump was installed at the bottom of each mesocosm to mimic natural water column disturbance, facilitating vertical water exchange and thorough mixing within the system. The experimental system was open; during inclement weather, a rainproof film was installed approximately 2.5 m above the ground.

The experiment comprised one control treatment and three nutrient addition treatments, each with three replicates. The control treatment received no nutrient supplementation (C). The nutrient addition treatments included sole nitrogen addition (N), sole phosphorus addition (P), and combined nitrogen and phosphorus addition (N + P). Given that algae can directly assimilate nitrate (NO_3_^−^) and phosphate (PO_4_^3−^), potassium nitrate (KNO_3_) and potassium dihydrogen phosphate (KH_2_PO_4_) were employed as the nitrogen and phosphorus sources, respectively. In the winter experiment, 0.219 g of KNO_3_ was added per application to the N and N + P treatments, while 0.017 g of KH_2_PO_4_ was added per application to the P and N + P treatments. In the spring experiment, 0.160 g of KNO_3_ and 0.012 g of KH_2_PO_4_ were added per application to the corresponding treatments. Nutrient additions were performed twice per week, with equal time intervals between applications.

Prior to the initiation of the experiment, raw water from the reservoir was pumped into a transfer tank and filtered through a 2 mm mesh to eliminate large debris, fish, and other large organisms. This coarse screening did not remove the natural phytoplankton or zooplankton communities. After thorough homogenization, the water was evenly distributed into the experimental mesocosms to ensure similar initial physicochemical conditions among all units before the experiment began. The initial concentrations of total phosphorus (TP) and total nitrogen (TN) in the raw water were 0.036 ± 0.002 mg/L and 0.799 ± 0.027 mg/L in winter and 0.046 ± 0.002 mg/L and 1.085 ± 0.013 mg/L in spring, respectively. Throughout the experiment, the water volume in each mesocosm was maintained at 800 L, with distilled water added as required to compensate for evaporative losses. During the experimental period, the inner walls and bottoms of the mesocosms were gently brushed three times per day using a soft-bristled brush to minimize the growth of periphytic algae. The dislodged algae were not physically removed from the mesocosms and could be resuspended into the water column. During sampling, resuspended material was collected together with the water-column phytoplankton. The algal community in our mesocosms was composed predominantly of planktonic taxa or taxa capable of both planktonic and attached growth, and no obligately periphytic taxa were identified.

### 2.2. Sample Collection and Analyses

Integrated water samples were collected to quantify TP, TN, and phytoplankton (community composition and biomass) responses. The water chemistry parameters measured in this experiment encompassed TP and TN. TP was measured using potassium persulfate digestion in combination with ammonium molybdate spectrophotometry (GB 11893-89 [51]), whereas TN was determined via alkaline potassium persulfate digestion followed by ultraviolet spectrophotometry (GB 11894-89 [52]). Samples for phytoplankton composition and biomass were preserved in Lugol’s solution. Phytoplankton was analyzed using the Utermöhl method, and volume was estimated from geometrical shape. Phytoplankton taxa were identified based on observable morphological characteristics using the identification book [53]. When species-level identification was uncertain, taxa were assigned to a higher taxonomic level based on the available morphological characteristics. It should be noted that Lugol’s iodine preservation may cause cell shrinkage, lysis of fragile flagellated taxa, and loss of diagnostic morphological features, particularly in groups such as Cryptophyta and Dinophyta. Therefore, some preservation-related bias in the abundance of fragile taxa cannot be completely excluded. However, because the subsequent community-level analyses were conducted at the phylum level and all samples were processed using the same preservation protocol, such bias is less likely to substantially affect the main conclusions of this study.

At the conclusion of the experiment, phytoplankton samples were collected for fatty acid analysis. The glass fiber filters (Whatman GF/F, pore size 0.7 μm, GE Healthcare, Little Chalfont, UK) employed for sample collection were pre-treated through combustion at 500 °C for 4 h in a muffle furnace, followed by rinsing in 10% (*v*/*v*) hydrochloric acid for 15 min, triple-washing with distilled water, and freeze-drying in a lyophilizer before use. Before phytoplankton sampling, the water from the experimental system was homogenized and pre-filtered through a 50 μm mesh, after which the filtrate was collected onto the pre-treated GF/F filters using a vacuum filtration system [54]. Consequently, particles and organisms smaller than 0.7 μm and those larger than 50 μm were excluded from the analysis. The filter samples were stored at −20 °C until fatty acid extraction.

After weighing, the freeze-dried phytoplankton samples were transferred into 10 mL glass centrifuge tubes. Each tube was filled with 2 mL of a methanol-to-toluene mixture containing 2% (*v*/*v*) concentrated sulfuric acid, with a methanol-to-toluene volume ratio of 9:1. Subsequently, 20 μL of an internal standard solution was added to each tube using a 25 μL microsyringe. The internal standard solution was prepared by dissolving 4.47 μL of methyl laurate (C12:0) in n-hexane: n-hexane was first added to a 50 mL glass volumetric flask, followed by the addition of methyl laurate using a 5 μL microsyringe, after which the solution was thoroughly mixed, resulting in a final internal standard concentration of 0.1 μg μL^−1^. The centrifuge tubes were flushed with nitrogen gas and immediately sealed, then placed in a magnetic stirrer preheated to 80 °C. The stirrer bath contained ultrapure water, and the rotation speed was set to 3000 r min^−1^. Samples were digested for 1.5 h under continuous magnetic stirring. After digestion, 1 mL of ultrapure water and 1 mL of n-hexane were added to each tube, followed by vigorous agitation on a laboratory shaker. The samples were then centrifuged at 3500 r min^−1^ for 10 min. The upper organic phase was carefully transferred to a new glass centrifuge tube using a glass Pasteur pipette and evaporated to dryness under a gentle nitrogen stream. The residue was redissolved in 1 mL of n-hexane and finally transferred into a 2 mL amber autosampler vial for analysis. Fatty acid composition and quantification were carried out using a single quadrupole gas chromatography–mass spectrometry system (TRACE GC–MS, Thermo Finnigan, San Jose, CA, USA).

### 2.3. Statistical Analyses

Calculate the phytoplankton diversity index (*H*′ Shannon–Wiener index) using the following formula:

(1) $$H^{\prime }=-\Sigma_{i=1}^{s}P_{i}ln\left(P_{i}\right)$$

*P_i_*: Ratio of the number of individuals of the *i*-th phylum to the total number of individuals.

FA analysis encompassed SAFA, MUFA, PUFA, total fatty acids (TFA), ω-3 PUFA, ω-6 PUFA, and the ω-3: ω-6 PUFA ratio. Specifically, SAFA was computed as the summation of C13:0, C14:0, C15:0, C16:0, C17:0, C18:0, C20:0, C21:0, and C22:0. MUFA was defined as the aggregate of C14:1ω5, C15:1ω5, C16:1ω9, C17:1ω9, C18:1ω8, C18:1ω9, C19:1ω9, C20:1ω9, and C22:1ω9. PUFA was calculated as the sum of C16:2ω6, C16:3ω3, C16:3ω6, C16:4ω3, C18:4ω3, C18:3ω6, C20:2ω6, C20:3ω6, C22:2ω6, C18:2ω6 (LIN), C18:3ω3 (ALA), C20:4ω6 (ARA), C20:5ω3 (EPA), and C22:6ω3 (DHA). ω-3 PUFA was defined as the sum of C16:3ω3, C16:4ω3, C18:4ω3, ALA, EPA, and DHA, while ω-6 PUFA consisted of the sum of C16:2ω6, C16:3ω6, C18:3ω6, C20:2ω6, C20:3ω6, C22:2ω6, LIN, and ARA. Additionally, ARA, EPA, DHA, LIN, and ALA were individually analyzed as key fatty acids.

Differences in TN and TP concentrations among nutrient treatments at the conclusion of the experiment were examined. One-way analysis of variance (one-way ANOVA) was employed when the assumptions of normality and homogeneity of variances were satisfied; otherwise, the Kruskal–Wallis test was utilized. Differences in fatty acid indices among treatments at the same sampling time were analyzed using the identical approach. Fatty acid data were natural-log transformed using ln(x) before analysis.

Differences in phytoplankton community structure among treatments were evaluated using non-metric multidimensional scaling (NMDS) based on phytoplankton phylum-level biomass data. Community dissimilarities among samples were calculated using Bray–Curtis distances. NMDS ordination was performed using the metaMDS function in the vegan package (version 2.7.2) in R (version 4.4.1), with two dimensions (k = 2), a maximum of 100 iterations, and a fixed random seed to guarantee reproducibility. The goodness-of-fit of the NMDS ordination was assessed using stress values. The statistical significance of differences in phytoplankton community structure among treatments was tested using permutational multivariate analysis of variance (PERMANOVA) based on the Bray–Curtis distance matrix, implemented with the adonis2 function in the vegan package (version 2.7.2) and 999 permutations. PERMANOVA results were presented as coefficients of determination (*R*^2^) and associated *p*-values.

To comprehensively assess the direct and indirect relationships among nutrient factors, phytoplankton, and ω-3 PUFA content, a pSEM was applied. The pSEM analysis was conducted using the piecewiseSEM R package (version 2.3.0.1). Before model construction, all continuous variables were standardized using a z-score transformation to minimize the influence of differences in measurement scales on model estimation. The pSEM consisted of multiple linear models, with ω-3 PUFA content designated as the final response variable. Nutrient factors, including TN, TP, and nitrogen and phosphorus enrichment treatments, were incorporated as exogenous variables, while phytoplankton community structure indicators (the Shannon–Wiener index and phytoplankton biomass) were treated as mediating variables. All hypothesized pathways were fitted using linear regression models and subsequently integrated into a structural equation model using the *psem* function. Overall model fit was evaluated using Fisher’s C statistic and its associated significance level, with *p* > 0.05 indicating an adequate fit between the model and the data. Standardized regression coefficients for each pathway were used to compare the relative strengths of different variables influencing ω-3 PUFA content. Moreover, an AIC-based stepwise regression procedure was applied to the ω-3 PUFA regression submodel to select explanatory variables, aiming to obtain a more parsimonious and robust optimal model.

## 3. Results

### 3.1. Nutrients and Temperature

**Figure 1:**
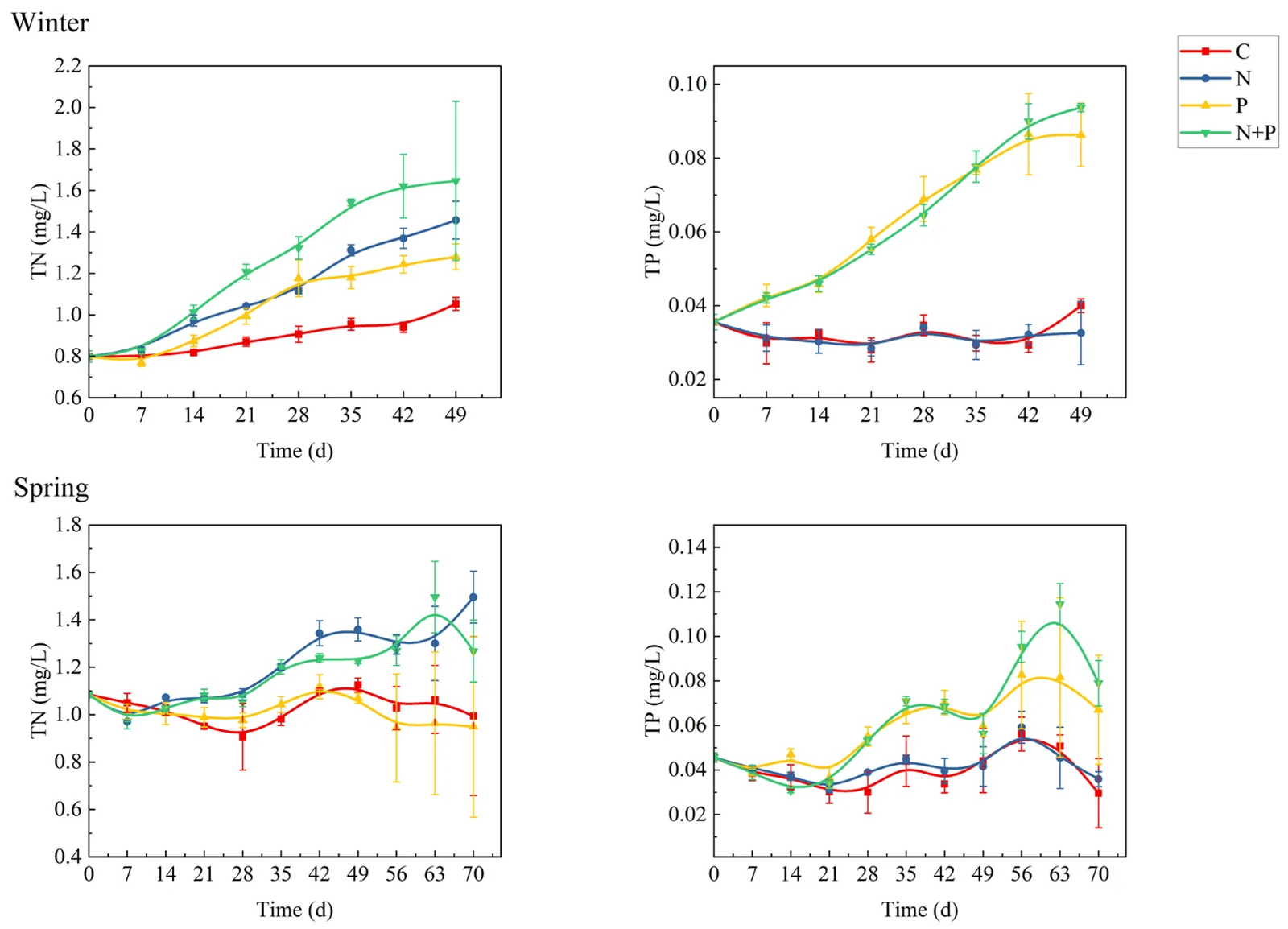
Temporal variations in total nitrogen (TN) and total phosphorus (TP) concentrations under different nutrient treatments.

During the winter experiment, the concentrations of TN and TP in the corresponding nutrient-enriched treatments increased steadily, while minor fluctuations were observed during the spring experiment (Figure 1). Overall, nutrient addition led to elevated TN and TP concentrations in the enriched treatments throughout the experiment.

At the end of the winter experiment, significant disparities among treatments were identified for both TN and TP concentrations (*p* < 0.05, Table 1). Conversely, at the end of the spring experiment, significant differences among treatments were only observed for TP concentrations (*p* < 0.05). The water temperature was higher in spring compared to winter, with a mean seasonal difference of 11.45 °C.

### 3.2. Phytoplankton Community Structure

**Figure 2:**
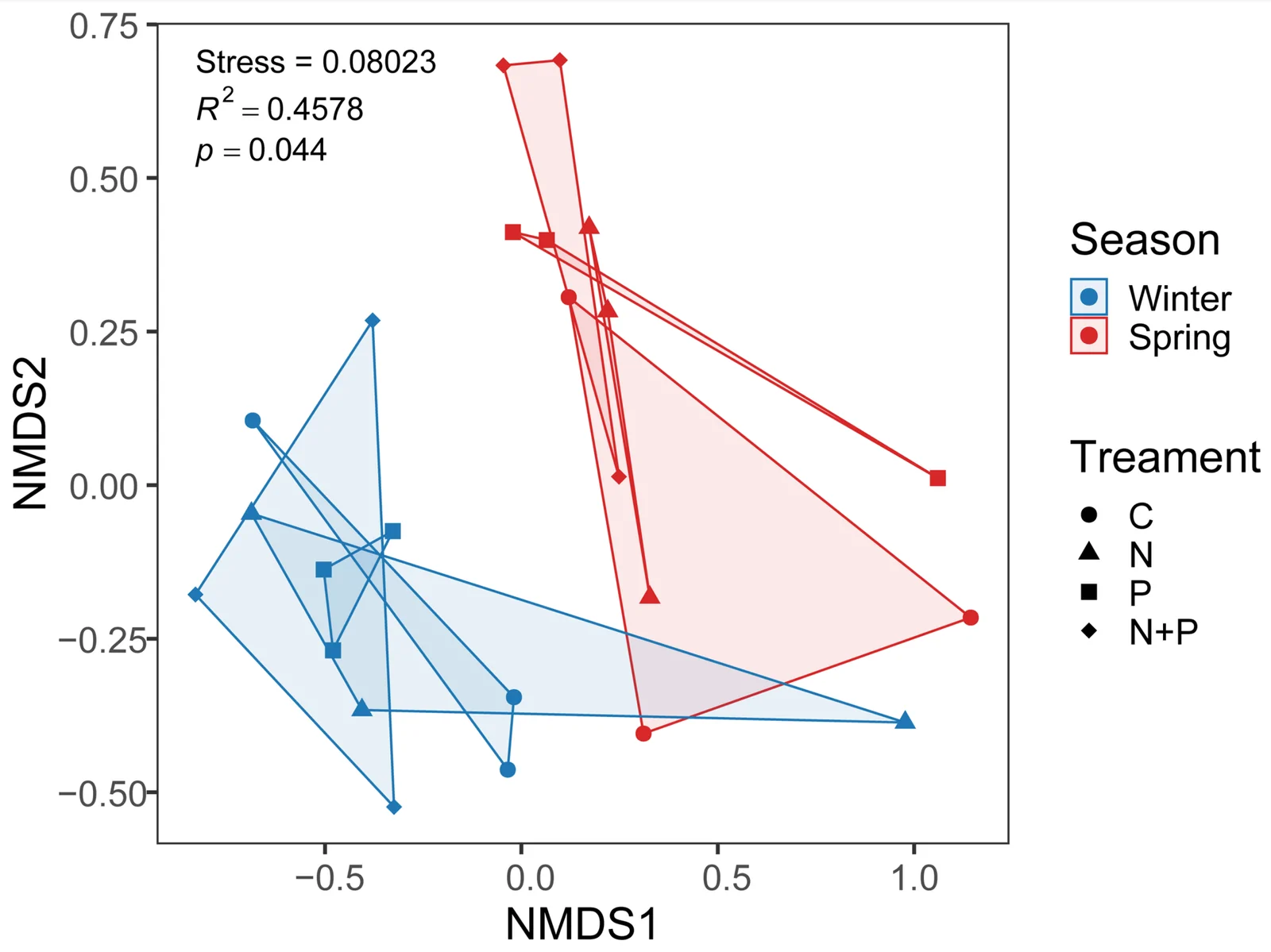
Non-metric multidimensional scaling (NMDS) analysis of phytoplankton community structure across seasons and nutrient treatments.

Upon the completion of the experiment, six phytoplankton phyla were identified, namely Cyanobacteriophyta, Chlorophyta, Cryptophyta, Dinophyta, Euglenophyta, and Bacillariophyta (Table S1). NMDS analysis (stress = 0.080) and PERMANOVA results demonstrated significant disparities in community composition (*p* < 0.05), which were predominantly influenced by seasonal variation. Specifically, notable differences were discerned between the winter and spring phytoplankton communities, while substantial overlap was observed among nutrient treatments within each season (Figure 2).

**Figure 3:**
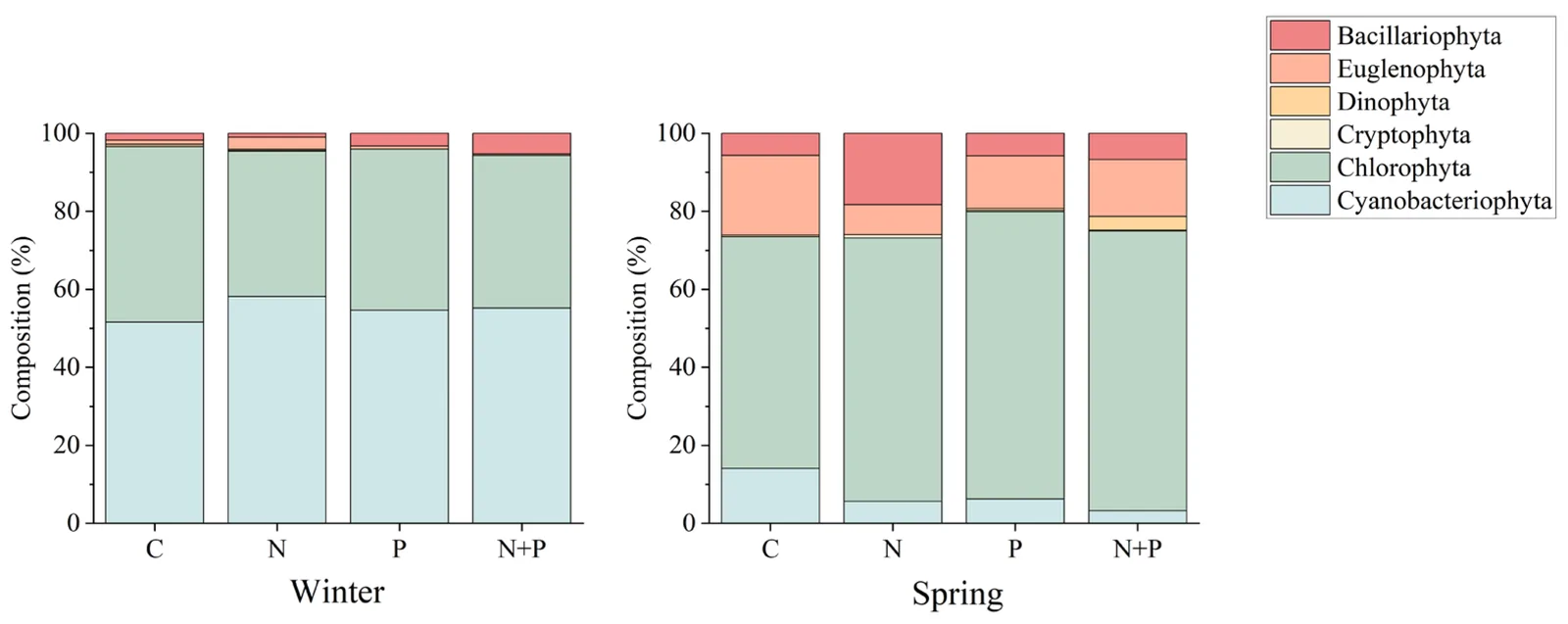
Relative biomass proportions of phytoplankton under different nutrient treatments.

In winter, the phytoplankton communities were predominantly composed of Cyanobacteriophyta and Chlorophyta, accounting for 52% to 58% and 37% to 45% of the total biomass, respectively. Bacillariophyta and Euglenophyta contributed relatively minor proportions, with non-cyanobacterial and non-chlorophyte phytoplankton collectively accounting for 3% to 6% of the total. Cryptophyta and Dinophyta were not detected in the P and N + P treatments. In spring, Chlorophyta dominated the phytoplankton community, accounting for 59% to 74% of the total biomass. The proportion of other phytoplankton groups (excluding Cyanobacteriophyta and Chlorophyta) was higher than that in winter, ranging from 20% to 27%. Cryptophyta were not observed in the control group, while Dinophyta were absent from the N treatment (Figure 3, Table S2).

The total phytoplankton biomass in winter was 22 ± 15 mg L^−1^, 21 ± 15 mg L^−1^, 25 ± 2 mg L^−1^, and 28 ± 10 mg L^−1^ across treatments, respectively. The corresponding values in spring were 11 ± 8 mg L^−1^, 16 ± 4 mg L^−1^, 16 ± 8 mg L^−1^, and 22 ± 8 mg L^−1^. No significant differences in total phytoplankton biomass were identified between any nutrient treatment and the control group (*p* > 0.05).

### 3.3. Variations in Fatty Acids

**Figure 4:**
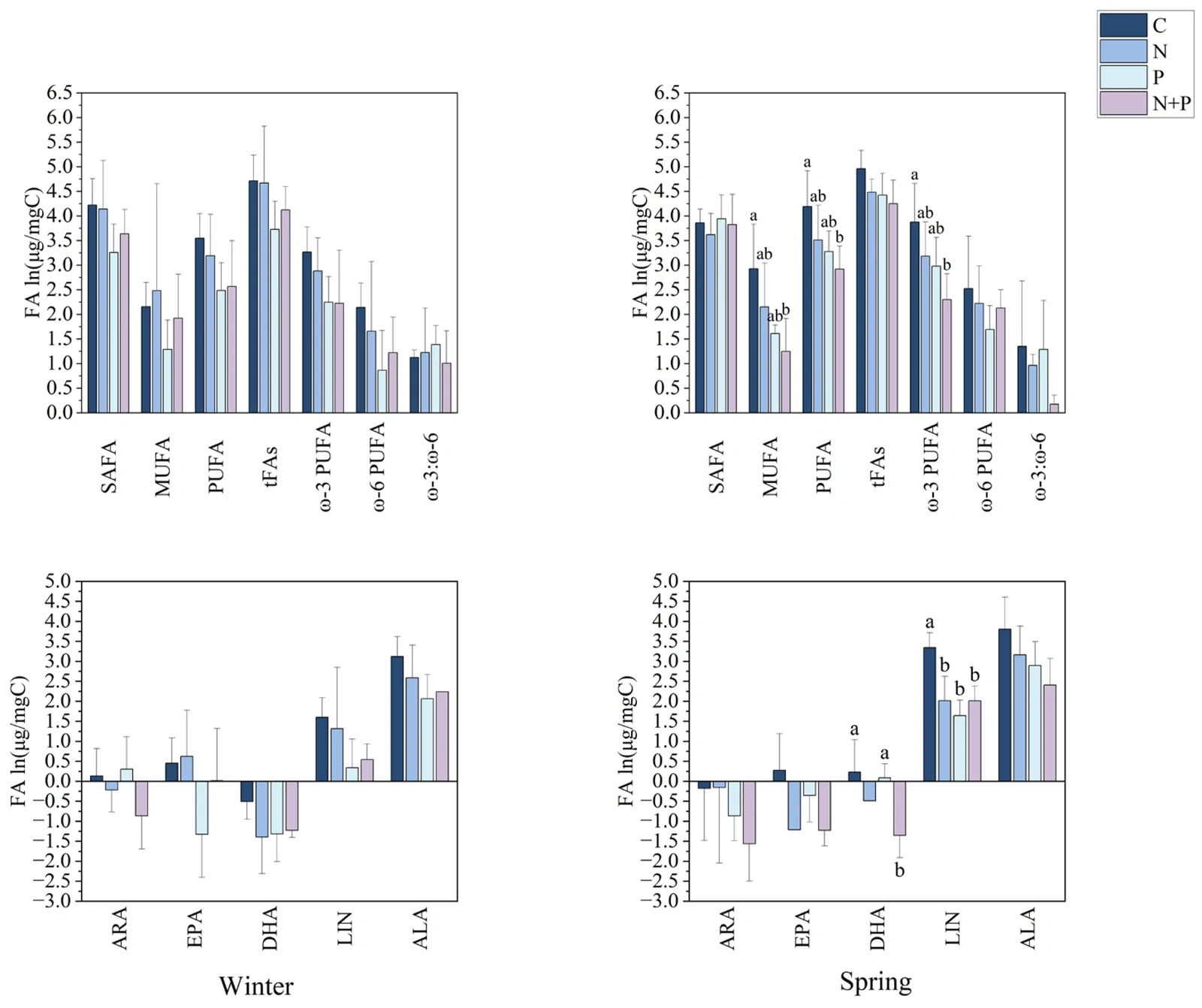
Contents of major fatty acids (FA) under different nutrient treatments. Different superscript letters indicate significant differences among treatments (*p* < 0.05), whereas values sharing at least one superscript letter are not significantly different.

No notable alterations in fatty acid content were detected following nutrient additions during the winter season. Conversely, during spring, nitrogen and phosphorus enrichment had more extensive impacts on fatty acids. The N + P treatment showed significantly lower levels of MUFA, PUFA, ω-3 PUFA, and DHA compared with the control. Additionally, the contents of linoleic acid (LIN) in the N, P, and N + P treatments were all significantly lower than those in the control (Figure 4).

PSEM was employed to assess the relationships among nutrient enrichment, phytoplankton biomass, diversity, and ω-3 PUFA in winter and spring. Both seasonal pSEMs passed the global goodness-of-fit tests (winter: Fisher’s C = 9.789, *p* = 0.280; spring: Fisher’s C = 1.269, *p* = 0.867), suggesting a good model fit. The winter model accounted for 58% of the variation in ω-3 PUFA (*R*^2^ = 0.58), while the explanatory power increased in spring, accounting for 76% of the variation (*R*^2^ = 0.76).

**Figure 5:**
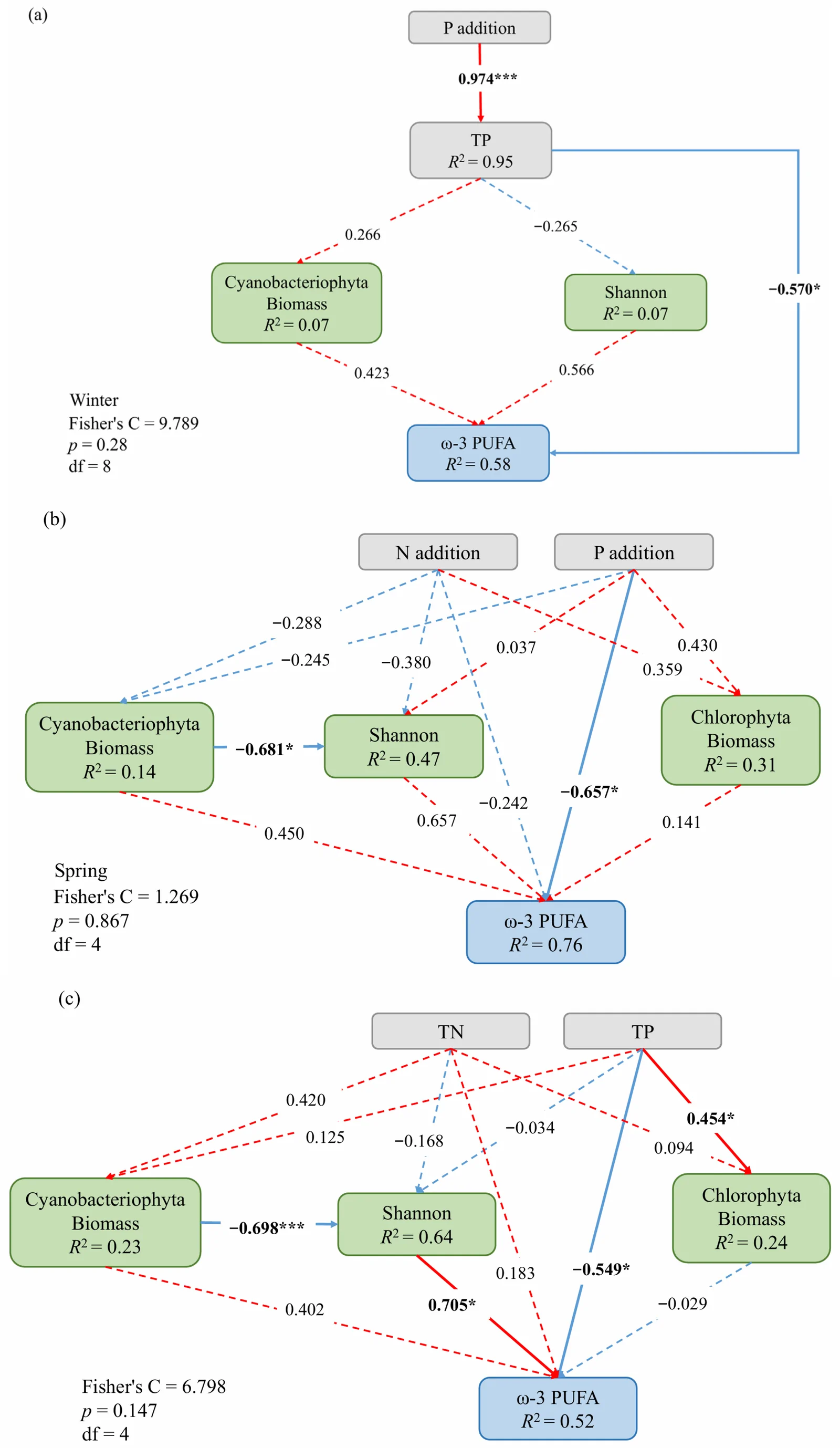
Piecewise structural equation modeling (pSEM) analysis of the effects of nutrient enrichment, phytoplankton biomass, and diversity on ω-3 PUFA, including season-specific models for winter (**a**) and spring (**b**), and a pooled model combining both seasons (**c**). (Red arrows indicate positive relationships, whereas blue arrows indicate negative relationships. Solid lines denote statistically significant effects, and dashed lines indicate non-significant effects. Numbers on arrows represent standardized path coefficients. Asterisks indicate significance levels: * indicates *p* < 0.05, and *** indicates *p* < 0.001.).

In winter, phosphorus addition significantly elevated TP concentrations, and TP had a significant direct negative effect on ω-3 PUFA. Cyanobacterial biomass and phytoplankton diversity (Shannon–Wiener index) had no significant effects on ω-3 PUFA, and TP did not significantly influence phytoplankton biomass or diversity (Figure 5a). In spring, phosphorus addition also significantly reduced ω-3 PUFA, whereas the pathways involving nitrogen addition, phytoplankton biomass, and phytoplankton diversity were not significant (Figure 5b). Nutrient additions had no significant effects on phytoplankton biomass or diversity. Moreover, the explanatory power for phytoplankton biomass and diversity was low in winter, while phytoplankton biomass showed low explanatory power in spring. Cyanobacterial biomass had a significant negative effect on phytoplankton diversity in spring, indicating that phytoplankton biomass and community structure may be more strongly regulated by non-nutrient factors. The consistency of pSEM results across winter and spring indicates that phosphorus is a key factor regulating ω-3 PUFA.

A pSEM analysis was carried out by integrating winter and spring data to evaluate the relationships among nutrient addition, phytoplankton biomass, diversity, and ω-3 PUFA (Figure 5c). This cross-seasonal pSEM passed the global goodness-of-fit test (Fisher’s C = 6.798, *p* = 0.147), indicating an adequate model fit. The model explained 52% of the variance in ω-3 PUFA (*R*^2^ = 0.52). Within this model, TP continued to have a significant negative effect on ω-3 PUFA and a significant positive effect on chlorophyte biomass, while having no significant effects on cyanobacterial biomass or phytoplankton diversity. Cyanobacterial biomass had a significant negative effect on phytoplankton diversity, whereas community diversity had a significant positive effect on ω-3 PUFA. Collectively, TP and phytoplankton community diversity jointly influenced ω-3 PUFA. The standardized direct effect of the Shannon–Wiener index on ω-3 PUFA was +0.705, whereas the standardized direct effect of TP was −0.549. Comparison of the absolute values of these coefficients indicated that the direct effect of community diversity on ω-3 PUFA was approximately 1.28 times stronger than that of TP.

## 4. Discussion

### 4.1. Seasonality Plays a Dominant Role in Phytoplankton Community Structure, Whereas Nutrient Enrichment Shows Limited Effects in This Study

In this study, nutrient enrichment did not exert a significant influence on the structure or biomass of the algal community. NMDS analysis revealed a distinct separation between winter and spring samples, while no clear separation was discerned among nutrient treatments. This implies that the addition of nitrogen and phosphorus had limited impacts on the phytoplankton community structure within this eutrophic system, whereas seasonal variation was highly prominent. This pattern is likely due to the reservoir already being in a eutrophic state, under which additional external nutrient inputs have only marginal effects on the system. Previous research has indicated that the response to nutrient enrichment is contingent upon the initial nutrient status of the system. In highly productive or eutrophic environments, the stimulatory effects are often significantly weakened [55]. Therefore, seasonal drivers remain the predominant factors shaping the phytoplankton community structure, a conclusion further corroborated by pSEM.

Cyanobacteria were the dominant component of the algal community in winter, while chlorophytes became dominant in spring. Eutrophication is widely acknowledged as a crucial driver of cyanobacterial dominance, with numerous studies demonstrating that nutrient enrichment promotes the prevalence of cyanobacteria or chlorophytes in phytoplankton communities [56,57,58,59].

Located in tropical and subtropical areas, the reservoir experienced winter water temperatures ranging from 18 to 26 °C, which remained within the temperature range suitable for cyanobacterial growth. In spring, phytoplankton communities were dominated by chlorophytes, accompanied by a significant decline in the relative biomass of cyanobacteria. With increasing light availability and rising temperatures (24–31 °C during the spring experiment), chlorophytes can reach or approach their maximum photosynthetic efficiency within the range of 25–30 °C [60,61], thereby attaining higher growth rates and competitive advantages under favorable thermal and light conditions. Concurrently, increased rainfall enhances water column turbulence and vertical mixing, which can weaken the competitive advantage of cyanobacteria in stable water bodies [62]. Increased nutrient availability through both external inputs and internal loading [63], may produce a hypereutrophic ecosystem that favors chlorophytes [58,59].

### 4.2. Phosphorus Availability Altered ω-3 PUFA Content of Basal Food Directly

Although nutrient addition did not significantly alter community structure, fatty acid analysis revealed marked changes in food quality. While winter nutrient addition did not induce significant changes in fatty acid content, spring responses became evident. MUFA, PUFA, ω-3 PUFA, and DHA levels decreased significantly in the N + P group, while LIN levels also decreased significantly in the N, P, and N + P groups. This result indicates that the system responds more sensitively to exogenous nutrients in spring and that nutrient addition can reduce the food quality of phytoplankton for aquatic consumers.

PSEM further revealed pathway relationships among nutrients, phytoplankton biomass, diversity, and ω-3 PUFA. Results indicated phosphorus influences ω-3 PUFA via a significant negative pathway, suggesting phosphorus is a key driver of phytoplankton nutritional quality. This aligns with other studies showing high phosphorus leads to a significant decline in ω-3 PUFA content [32]. However, in those studies, the decrease in ω-3 PUFA content following phosphorus enrichment was primarily attributed to alterations in phytoplankton community structure, where cyanobacteria lacking ω-3 PUFA synthesis became the dominant phytoplankton group, while the proportion of groups with high ω-3 PUFA synthesis capacity, such as diatoms, dinoflagellates, and cryptophytes, decreased [33,36]. In contrast, phosphorus addition to this eutrophic water body did not markedly alter phytoplankton community structure nor significantly affect phytoplankton diversity. This result may relate to the initially high phosphorus concentration in the reservoir. Consequently, phosphorus may reduce ω-3 PUFA levels by directly affecting phytoplankton physiological and biochemical responses rather than through community structure.

The biosynthesis of ω-3 PUFA involves a series of enzymatic reactions, including fatty acid desaturases and elongases, which introduce double bonds into fatty acid chains and extend their carbon length [64]. Previous studies have shown that phosphorus availability can regulate the transcription or expression of desaturases and elongases in algae [48,49], thereby influencing ω-3 PUFA content [46,47]. Lopes reported that under high-phosphorus conditions, the transcription levels of two key enzymes involved in ω-3 PUFA biosynthesis, Δ5 desaturase and Δ6 desaturase, were significantly downregulated during the exponential growth phase [65]. Similarly, Ahmad found that elevated phosphorus significantly suppressed the expression of ω-6 FAD and ω-3 FAD genes in *Chlorella*, with a more pronounced inhibitory effect observed for ω-3 FAD [66]. These previous findings provided theoretical support for our pSEM analysis. However, we did not directly measure the expression or activity of desaturases, elongases, or other enzymes involved in fatty acid biosynthesis. Thus, our results suggest a potential physiological contribution to the observed decline in ω-3 PUFA, rather than providing direct physiological evidence for a specific biochemical mechanism.

In addition, phytoplankton functional traits include cell size, motility, phosphate affinity, and other related traits [67]. Changes in phosphorus availability may affect phytoplankton functional traits and their associated physiological processes without necessarily causing detectable shifts in taxonomic composition. Previous studies have shown that variation in phosphorus availability can influence phytoplankton cell size, phosphorus uptake or intracellular phosphorus storage, and photosynthetic processes [68,69,70]. Such responses may also affect cellular fatty acid biosynthesis. However, because these functional traits and associated cellular physiological processes were not directly measured in the study, their contribution to the changes in ω-3 PUFA under phosphorus enrichment may be regarded as a potential mechanism.

### 4.3. Phosphorus and Algal Diversity Jointly Regulate ω-3 PUFA Composition via Independent Pathways

When winter and spring data were combined for pSEM, TP still exhibited a significant negative effect on ω-3 PUFA, while no significant effect was observed on algal diversity. In contrast, the Shannon diversity index showed a significant positive effect on ω-3 PUFA, indicating that phytoplankton community diversity is also an important factor influencing fatty acid composition. These results suggest that in the present system, phosphorus concentration and algal diversity jointly influence ω-3 PUFA through independent pathways, although the underlying mechanisms differ between the two drivers.

The influence of algal diversity on fatty acid composition primarily arises from taxonomic differences in fatty acid profiles. Previous studies have shown that fatty acid responses to environmental change vary in both direction and magnitude among species, with the contents of EPA and DHA being largely determined by species identity [71]. Taxonomic differences can explain 56–84% of the variation in key PUFA indicators [72], and their influence has even been reported to be 3–4 times that of the most important growth-related environmental variables [24]. *H*′ was used in this study to characterize phytoplankton community diversity and structure, rather than as a direct measure of nutritional quality. Although *H*′ captures the diversity and relative distribution of taxa, it does not account for taxon-specific differences in fatty acid composition and nutritional value. To evaluate whether particular taxonomic groups provided additional explanatory information, the biomass of all six phytoplankton taxonomic groups was initially considered as candidate predictors in the pSEM. However, the biomass of EPA/DHA-rich taxa was not retained during AIC-based model selection, indicating that these variables did not provide sufficient additional explanatory information for ω-3 PUFA variation within the fitted model. Their relatively low biomass in communities dominated by Cyanobacteriophyta and Chlorophyta may have limited their contribution to variation in the overall ω-3 PUFA pool. Thus, *H*′ in our model should be interpreted as a general indicator of community diversity rather than a direct proxy for nutritional quality. Future studies with greater representation of nutritionally important taxa could use taxon-specific metrics, such as the biomass or relative biomass proportion of EPA/DHA-rich taxa, to more directly evaluate their contribution to food quality.

At the community level, different algal taxa vary in their fatty acid synthesis capacity, enzymatic regulation, and resource utilization strategies. These differences, together with their interactions, jointly determine the final fatty acid composition, reflecting an integrated metabolic response at the community scale under multispecies coexistence. Although algal community diversity exerts a positive effect on ω-3 PUFA, the present results indicate that phosphorus also shows a direct negative regulatory effect on ω-3 PUFA. Within each seasonal experiment, the experimental treatments were conducted under the same prevailing environmental conditions, and the primary difference among treatments was the nutrient addition. Although phosphorus was the manipulated nutrient in our experiment, water temperature and light availability naturally differed between the winter and spring experimental runs and may have influenced algal growth, lipid metabolism, and ω-3 PUFA biosynthesis [73,74]. In addition, the availability of micronutrients can affect algal physiological processes, potentially contributing to variation in seston nutritional quality [75,76,77]. Because these environmental factors were not independently manipulated or comprehensively quantified in the study, their relative contributions to the observed ω-3 PUFA responses could not be directly assessed. Future studies that experimentally manipulate phosphorus together with temperature, light availability, or other relevant factors would be valuable for disentangling their individual and interactive effects on ω-3 PUFA.

## 5. Conclusions

By conducting nutrient-manipulated field mesocosm experiments utilizing natural plankton communities from a tropical eutrophic reservoir, this study showed that phosphorus enrichment exerts a direct negative effect on ω-3 PUFA content in phytoplankton without the interference of community changes. The decline in ω-3 PUFA is likely associated with phosphorus-induced metabolic regulation within algal cells. These findings enhance our comprehension of the fundamental mechanism by which nutrient loading impacts the aquatic food web and aquaculture from a nutritional dynamic perspective.

## Figures and Tables

**Table 1:** Water chemistry parameters under different nutrient treatments at the end of the experiment.

Season	Treatment	TP (mg/L)	*p*-Value	TN (mg/L)	*p*-Value	Temperature (°C)
Winter	Control	0.040 ± 0.002 ^ab^	**0.022**	1.053 ± 0.031 ^a^	**0.033**	18.30 ± 1.56
	N	0.033 ± 0.009 ^a^		1.456 ± 0.091 ^b^		
	P	0.086 ± 0.009 ^b^		1.280 ± 0.063 ^ab^		
	N + P	0.094 ± 0.001 ^b^		1.646 ± 0.384 ^b^		
Spring	Control	0.030 ± 0.016 ^a^	**0.012**	0.994 ± 0.335	0.066	29.75 ± 0.92
	N	0.036 ± 0.003 ^a^		1.496 ± 0.109		
	P	0.067 ± 0.024 ^ab^		0.949 ± 0.382		
	N + P	0.079 ± 0.010 ^b^		1.268 ± 0.131		

Different superscript letters indicate significant differences among treatments (*p* < 0.05), whereas values sharing at least one superscript letter are not significantly different. Bold values indicate statistically significant differences among treatments (*p* < 0.05).

## Data Availability

The data that support the findings of this study are available from the corresponding authors upon reasonable request.

## Supplementary Materials

The following supporting information can be downloaded at https://www.mdpi.com/article/10.3390/microorganisms14092060/s1, Figure S1. Field photograph of the experimental mesocosm setup; Table S1: Occurrence of phytoplankton taxa across different nutrient treatments. “+” indicates that the taxon was detected in the corresponding treatment; Table S2: Relative biomass proportions of phytoplankton under different nutrient treatments.

## Abbreviations

The following abbreviations are used in this manuscript:

EPA	Eicosapentaenoic acid
ALA	α-linolenic acid
ARA	Arachidonic acid
DHA	Docosahexaenoic acid
FA	Fatty acid
FAD	Fatty acid desaturase
LIN	Linoleic acid
MUFAs	Monounsaturated fatty acids
NMDS	Non-metric multidimensional scaling
One-way ANOVA	One-way analysis of variance
PERMANOVA	Permutational multivariate analysis of variance
pSEM	Piecewise structural equation modeling
PUFAs	Polyunsaturated fatty acids
SAFAs	Saturated fatty acids
TFAs	Total fatty acids
TN	Total nitrogen
TP	Total phosphorus
ω-3 PUFAs	Omega-3 polyunsaturated fatty acids

## References

[1] Brett M., Müller-Navarra D. (1997). The role of highly unsaturated fatty acids in aquatic foodweb processes. Freshw. Biol..

[2] Elser J.J., Fagan W.F., Denno R.F., Dobberfuhl D.R., Folarin A., Huberty A., Interlandi S., Kilham S.S., McCauley E., Schulz K.L. (2000). Nutritional constraints in terrestrial and freshwater food webs. Nature.

[3] Danielsdottir M.G., Brett M.T., Arhonditsis G.B. (2007). Phytoplankton food quality control of planktonic food web processes. Hydrobiologia.

[4] Pilecky M., Závorka L., Arts M.T., Kainz M.J. (2021). Omega-3 PUFA profoundly affect neural, physiological, and behavioural competences—Implications for systemic changes in trophic interactions. Biol. Rev..

[5] Yan K., Guo F., Kainz M.J., Li F., Gao W., Bunn S.E., Zhang Y. (2024). The importance of omega-3 polyunsaturated fatty acids as high-quality food in freshwater ecosystems with implications of global change. Biol. Rev..

[6] Monroig Ó., Shu-Chien A.C., Kabeya N., Tocher D.R., Castro L.F.C. (2022). Desaturases and elongases involved in long-chain polyunsaturated fatty acid biosynthesis in aquatic animals: From genes to functions. Prog. Lipid Res..

[7] Das U.N. (2006). Essential fatty acids: Biochemistry, physiology and pathology. Biotechnol. J..

[8] Heckmann L.-H., Sibly R.M., Timmermans M.J., Callaghan A. (2008). Outlining eicosanoid biosynthesis in the crustacean Daphnia. Front. Zool..

[9] Hulbert A.J., Kelly M.A., Abbott S.K. (2014). Polyunsaturated fats, membrane lipids and animal longevity. J. Comp. Physiol. B.

[10] Brett M.T., Kainz M.J., Taipale S.J., Seshan H. (2009). Phytoplankton, not allochthonous carbon, sustains herbivorous zooplankton production. Proc. Natl. Acad. Sci. USA.

[11] Butts I.A.E., Baeza R., Støttrup J.G., Krüger-Johnsen M., Jacobsen C., Pérez L., Asturiano J.F., Tomkiewicz J. (2015). Impact of dietary fatty acids on muscle composition, liver lipids, milt composition and sperm performance in European eel. Comp. Biochem. Physiol. Part A Mol. Integr. Physiol..

[12] Yanes-Roca C., Rhody N., Nystrom M., Main K.L. (2009). Effects of fatty acid composition and spawning season patterns on egg quality and larval survival in common snook (*Centropomus undecimalis*). Aquaculture.

[13] Liu J., Ma Q., Zhang F., Gao Q., Zhang Z., Wei Y., Liang M., Xu H. (2025). Dietary DHA regulated the androgen production in male Chinese tongue sole *Cynoglossus semilaevis*. Aquac. Nutr..

[14] Luo Y., Wang Y., Guo F., Kainz M.J., You J., Li F., Gao W., Shen X., Tao J., Zhang Y. (2024). Sources and fate of omega-3 polyunsaturated fatty acids in a highly eutrophic lake. Sci. Total Environ..

[15] Závorka L., Blanco A., Chaguaceda F., Cucherousset J., Killen S.S., Liénart C., Mathieu-Resuge M., Němec P., Pilecky M., Scharnweber K. (2023). The role of vital dietary biomolecules in eco-evo-devo dynamics. Trends Ecol. Evol..

[16] Taipale S.J., Rigaud C., Calderini M.L., Asikainen H., Litmanen J.J., Vesamäki J.S., Ndebele-Murisa M.R., Nhiwatiwa T. (2025). Production and transfer of essential fatty acids in a man-made tropical lake ecosystem. Limnol. Oceanogr..

[17] Gladyshev M.I., Sushchik N.N., Makhutova O.N. (2013). Production of EPA and DHA in aquatic ecosystems and their transfer to the land. Prostaglandins Other Lipid Mediat..

[18] Guo F., Ebm N., Bunn S.E., Brett M.T., Hager H., Kainz M.J. (2021). Longitudinal variation in the nutritional quality of basal food sources and its effect on invertebrates and fish in subalpine rivers. J. Anim. Ecol..

[19] Ahlgren G., Lundstedt L., Brett M., Forsberg C. (1990). Lipid composition and food quality of some freshwater phytoplankton for cladoceran zooplankters. J. Plankton Res..

[20] Ahlgren G., Gustafsson I.-B., Boberg M. (1992). Fatty acid content and chemical composition of freshwater microalgae. J. Phycol..

[21] Gulati R., Demott W. (1997). The role of food quality for zooplankton: Remarks on the state-of-the-art, perspectives and priorities. Freshw. Biol..

[22] Taipale S., Strandberg U., Peltomaa E., Galloway A., Ojala A., Brett M. (2013). Fatty acid composition as biomarkers of freshwater microalgae: Analysis of 37 strains of microalgae in 22 genera and in seven classes. Aquat. Microb. Ecol..

[23] Peltomaa E.T., Aalto S.L., Vuorio K.M., Taipale S.J. (2017). The importance of phytoplankton biomolecule availability for secondary production. Front. Ecol. Evol..

[24] Galloway A.W.E., Winder M. (2015). Partitioning the Relative Importance of Phylogeny and Environmental Conditions on Phytoplankton Fatty Acids. PLoS ONE.

[25] Schindler D.W., Hecky R.E., Findlay D.L., Stainton M.P., Parker B.R., Paterson M.J., Beaty K.G., Lyng M., Kasian S.E.M. (2008). Eutrophication of lakes cannot be controlled by reducing nitrogen input: Results of a 37-year whole-ecosystem experiment. Proc. Natl. Acad. Sci. USA.

[26] Conley D.J., Paerl H.W., Howarth R.W., Boesch D.F., Seitzinger S.P., Havens K.E., Lancelot C., Likens G.E. (2009). Controlling Eutrophication: Nitrogen and Phosphorus. Science.

[27] Paerl H.W., Scott J.T., McCarthy M.J., Newell S.E., Gardner W.S., Havens K.E., Hoffman D.K., Wilhelm S.W., Wurtsbaugh W.A. (2016). It takes two to tango: When and where dual nutrient (N & P) reductions are needed to protect lakes and downstream ecosystems. Environ. Sci. Technol..

[28] Finkel Z.V., Beardall J., Flynn K.J., Quigg A., Rees T.A.V., Raven J.A. (2010). Phytoplankton in a changing world: Cell size and elemental stoichiometry. J. Plankton Res..

[29] Edwards K.F., Thomas M.K., Klausmeier C.A., Litchman E. (2012). Allometric scaling and taxonomic variation in nutrient utilization traits and maximum growth rate of phytoplankton. Limnol. Oceanogr..

[30] Edwards K.F., Klausmeier C.A., Litchman E. (2015). Nutrient utilization traits of phytoplankton. Ecology.

[31] Müller-Navarra D.C., Brett M.T., Liston A.M., Goldman C.R. (2000). A highly unsaturated fatty acid predicts carbon transfer between primary producers and consumers. Nature.

[32] Müller-Navarra D.C., Brett M.T., Park S., Chandra S., Ballantyne A.P., Zorita E., Goldman C.R. (2004). Unsaturated fatty acid content in seston and tropho-dynamic coupling in lakes. Nature.

[33] Taipale S.J., Vuorio K., Strandberg U., Kahilainen K.K., Järvinen M., Hiltunen M., Peltomaa E., Kankaala P. (2016). Lake eutrophication and brownification downgrade availability and transfer of essential fatty acids for human consumption. Environ. Int..

[34] Trommer G., Lorenz P., Lentz A., Fink P., Stibor H. (2019). Nitrogen enrichment leads to changing fatty acid composition of phytoplankton and negatively affects zooplankton in a natural lake community. Sci. Rep..

[35] Keva O., Taipale S.J., Hayden B., Thomas S.M., Vesterinen J., Kankaala P., Kahilainen K.K. (2021). Increasing temperature and productivity change biomass, trophic pyramids and community-level omega-3 fatty acid content in subarctic lake food webs. Glob. Change Biol..

[36] Calderini M.L., Kahilainen K.K., Estlander S., Peltomaa E., Piro A.J., Rigaud C., Ruuhijärvi J., Salmi P., Vesterinen J., Vuorio K. (2023). Eutrophication effect on production and transfer of omega-3 fatty acids in boreal lake food webs. Sci. Total Environ..

[37] Ahlgren G., Hyenstrand P. (2003). Nitrogen limitation effects of different nitrogen sources on nutritional quality of two freshwater organisms, *Scenedesmus quadricauda* (Chlorophyceae) and *Synechococcus* sp. (Cyanophyceae). J. Phycol..

[38] Rasdi N.W., Qin J.G. (2014). Effect of N:P ratio on growth and chemical composition of *Nannochloropsis oculata* and *Tisochrysis lutea*. J. Appl. Phycol..

[39] Su G., Jiao K., Li Z., Guo X., Chang J., Ndikubwimana T., Sun Y., Zeng X., Lu Y., Lin L. (2016). Phosphate limitation promotes unsaturated fatty acids and arachidonic acid biosynthesis by microalgae *Porphyridium purpureum*. Bioprocess Biosyst. Eng..

[40] Vijayan J., Alvarez S., Naldrett M.J., Morse W., Maliva A., Wase N., Riekhof W.R. (2024). Nitrogen starvation leads to TOR kinase-mediated downregulation of fatty acid synthesis in the algae *Chlorella sorokiniana* and *Chlamydomonas reinhardtii*. BMC Plant Biol..

[41] Zhang L., Liu J. (2016). Enhanced fatty acid accumulation in *Isochrysis galbana* by inhibition of the mitochondrial alternative oxidase pathway under nitrogen deprivation. Bioresour. Technol..

[42] Huerlimann R., Steinig E.J., Loxton H., Zenger K.R., Jerry D.R., Heimann K. (2014). Effects of growth phase and nitrogen starvation on expression of fatty acid desaturases and fatty acid composition of *Isochrysis* aff. *galbana* (TISO). Gene.

[43] Spijkerman E., Wacker A. (2011). Interactions between P-limitation and different C conditions on the fatty acid composition of an extremophile microalga. Extremophiles.

[44] Mühlroth A., Winge P., El Assimi A., Jouhet J., Maréchal E., Hohmann-Marriott M.F., Vadstein O., Bones A.M. (2017). Mechanisms of phosphorus acquisition and lipid class remodeling under P limitation in a marine microalga. Plant Physiol..

[45] Alipanah L., Winge P., Rohloff J., Najafi J., Brembu T., Bones A.M. (2018). Molecular adaptations to phosphorus deprivation and comparison with nitrogen deprivation responses in the diatom *Phaeodactylum tricornutum*. PLoS ONE.

[46] Chen G., Qu S., Wang Q., Bian F., Peng Z., Zhang Y., Ge H., Yu J., Xuan N., Bi Y. (2014). Transgenic expression of delta-6 and delta-15 fatty acid desaturases enhances omega-3 polyunsaturated fatty acid accumulation in *Synechocystis* sp. PCC6803. Biotechnol. Biofuels.

[47] Norashikin M.N., Loh S.H., Aziz A., San Cha T. (2018). Metabolic engineering of fatty acid biosynthesis in *Chlorella vulgaris* using an endogenous omega-3 fatty acid desaturase gene with its promoter. Algal Res..

[48] Yang F., Xiang W., Li T., Long L. (2018). Transcriptome analysis for phosphorus starvation-induced lipid accumulation in *Scenedesmus* sp.. Sci. Rep..

[49] Shi Y., Liu M., Ding W., Liu J. (2020). Novel insights into phosphorus deprivation boosted lipid synthesis in the marine alga *Nannochloropsis oceanica* without compromising biomass production. J. Agric. Food Chem..

[50] Zhang L., Wang N., Yang M., Ding K., Wang Y.-Z., Huo D., Hou C. (2019). Lipid accumulation and biodiesel quality of *Chlorella pyrenoidosa* under oxidative stress induced by nutrient regimes. Renew. Energy.

[51] (1989). Water Quality-Determination of Total Phosphorus-Ammonium Molybdate Spectrophotometric Method.

[52] (1989). Water Quality-Determination of Total Nitrogen-Alkaline Potassium Persulfate Digestion-UV Spectrophotometric Method.

[53] Xu Y., Xiao L., Yang Y. (2020). Common Phytoplankton Species Diversity and Atlas of Reservoirs in Zhuhai (in Chinese).

[54] Taipale S.J., Ventelä A.-M., Litmanen J., Anttila L. (2022). Poor nutritional quality of primary producers and zooplankton driven by eutrophication is mitigated at upper trophic levels. Ecol. Evol..

[55] Schulhof M.A., Shurin J.B., Declerck S.A.J., Van de Waal D.B. (2019). Phytoplankton growth and stoichiometric responses to warming, nutrient addition and grazing depend on lake productivity and cell size. Glob. Change Biol..

[56] Smith V.H. (2003). Eutrophication of freshwater and coastal marine ecosystems a global problem. Environ. Sci. Pollut. Res..

[57] Huisman J., Codd G.A., Paerl H.W., Ibelings B.W., Verspagen J.M.H., Visser P.M. (2018). Cyanobacterial blooms. Nat. Rev. Microbiol..

[58] Jensen J.P., Jeppesen E., Olrik K., Kristensen P. (1994). Impact of nutrients and physical factors on the shift from cyanobacterial to chlorophyte dominance in shallow Danish lakes. Can. J. Fish. Aquat. Sci..

[59] Karpowicz M., Zieliński P., Grabowska M., Ejsmont-Karabin J., Kozłowska J., Feniova I. (2020). Effect of eutrophication and humification on nutrient cycles and transfer efficiency of matter in freshwater food webs. Hydrobiologia.

[60] Yokohama Y. (1973). A comparative study on photosynthesis temperature relationships and their seasonal changes in marine benthic algae. Int. Rev. Gesamten Hydrobiol. Hydrogr..

[61] Benavides A.M.S., Jiménez-Conejo N., Torzillo G. (2026). Synergistic effect of light and temperature on growth and biochemical composition of *Chlorella sorokiniana* cultures. Foods.

[62] Bai Y., Huang T. (2025). Effects of artificial mixing on phytoplankton in a warm stratified drinking water reservoir: Characterization, mechanism, and implication. Water Res..

[63] Mohammed A., Mengistou S., Fetahi T. (2023). Role of environmental variables and seasonal mixing in dynamics of the phytoplankton community in a Tropical Highland Lake Ardibo, Ethiopia. J. Freshw. Ecol..

[64] Mühlroth A., Li K., Røkke G., Winge P., Olsen Y., Hohmann-Marriott M., Vadstein O., Bones A. (2013). Pathways of lipid metabolism in marine algae, co-expression network, bottlenecks and candidate genes for enhanced production of EPA and DHA in species of Chromista. Mar. Drugs.

[65] Lopes R.G., Cella H., Mattos J.J., Marques M.R.F., Soares A.T., Antoniosi Filho N.R., Derner R.B., Rörig L.R. (2019). Effect of phosphorus and growth phases on the transcription levels of EPA biosynthesis genes in the diatom *Phaeodactylum tricornutum*. Braz. J. Bot..

[66] Ahmad A., Osman S.M., Cha T.S., Loh S.H. (2016). Phosphate-induced changes in fatty acid biosynthesis in *Chlorella* sp. KS-MA2 strain. BioTechnologia.

[67] Wentzky V.C., Tittel J., Jäger C.G., Bruggeman J., Rinke K. (2020). Seasonal succession of functional traits in phytoplankton communities and their interaction with trophic state. J. Ecol..

[68] Peter K.H., Sommer U. (2015). Interactive effect of warming, nitrogen and phosphorus limitation on phytoplankton cell size. Ecol. Evol..

[69] Yan P., Guo J.-s., Zhang P., Xiao Y., Li Z., Zhang S.-q., Zhang Y.-x., He S.-x. (2021). The role of morphological changes in algae adaptation to nutrient stress at the single-cell level. Sci. Total Environ..

[70] Raj S., Sreenikethanam A., Gobi M., Sakate D., Jayakumar T., Rakesh S., Banu J.R., Bajhaiya A.K. (2025). Effect of phosphate availability on the dynamics of polyphosphate accumulation in microalgae. Sci. Rep..

[71] Calderini M.L., Pääkkönen S., Salmi P., Peltomaa E., Taipale S.J. (2023). Temperature, phosphorus and species composition will all influence phytoplankton production and content of polyunsaturated fatty acids. J. Plankton Res..

[72] Taipale S., Peltomaa E., Salmi P. (2020). Variation in ω-3 and ω-6 polyunsaturated fatty acids produced by different phytoplankton taxa at early and late growth phase. Biomolecules.

[73] Hixson S.M., Arts M.T. (2016). Climate warming is predicted to reduce omega-3, long-chain, polyunsaturated fatty acid production in phytoplankton. Glob. Change Biol..

[74] Guihéneuf F., Stengel D.B. (2017). Interactive effects of light and temperature on pigments and n-3 LC-PUFA-enriched oil accumulation in batch-cultivated *Pavlova lutheri* using high-bicarbonate supply. Algal Res..

[75] Chen X., Wakeham S.G., Fisher N.S. (2011). Influence of iron on fatty acid and sterol composition of marine phytoplankton and copepod consumers. Limnol. Oceanogr..

[76] Makareviciute-Fichtner K., Matthiessen B., Lotze H.K., Sommer U. (2021). Phytoplankton nutritional quality is altered by shifting Si: N ratios and selective grazing. J. Plankton Res..

[77] Wang H., Su Q., Zhuang Y., Wu C., Tong S., Guan B., Zhao Y., Qiao H. (2023). Effects of iron valence on the growth, photosynthesis, and fatty acid composition of *Phaeodactylum tricornutum*. J. Mar. Sci. Eng..

