# Supplementary material for: Intraspecific Physiological Alterations in Phytoplankton Declined the Food Nutritional Quality in Tropical Nutrient-Manipulated Aquatic Mesocosms
Source: Microorganisms. 2026 Sep 15;14(9):2060. doi: 10.3390/microorganisms14092060 (PMC13609077; doi:10.3390/microorganisms14092060)
Supplement: Supplementary file 1 [file microorganisms-14-02060-s001.zip › microorganisms-4472237-supplementary.pdf]

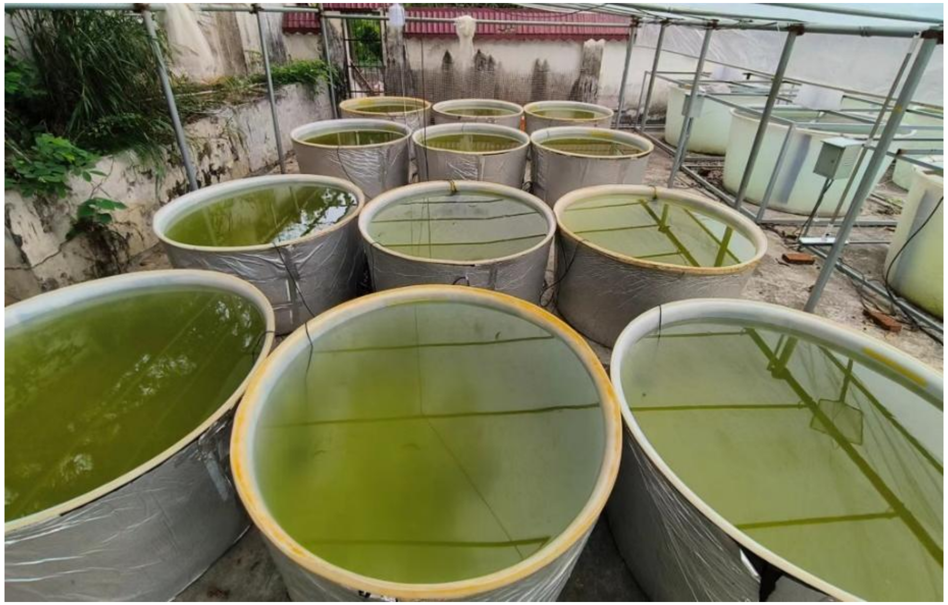

Figure S1. Field photograph of the experimental mesocosm setup.

Table S1. Occurrence of phytoplankton taxa across different nutrient treatments. "+" indicates that the taxon was detected in the corresponding treatment.

		Winter				Spring			
Phylum	Species	C	N	P	N+P	C	N	P	N+P
Cyanobacteriophyta	*Aphanocapsa elachista*	+	+	+	+	+	+	+	+
	Chroococcus limneticus	+	+			+	+	+	
	Chroococcus turgidus						+	+	
	Microcystis sp.					+	+		+
	Merismopedia minima						+	+	+
	Pseudanabaena limnetica						+		
	Pseudanabaena catenata					+	+		
	Lyngbya limnetica					+	+		+
	Limnothrix sp.		+	+	+	+	+	+	+

	Cylindrospermopsis raciborskii	+	+	+	+				
	Dolichospermum sp.				+			+	+
	Cuspidothrix issatschenkoi			+				+	
Chlorophyta	*Scenedesmus bijuga*	+	+	+	+	+	+	+	+
	Tetraedron pusillum	+	+	+	+	+	+	+	+
	Tetraedron caudatum	+	+	+	+	+	+		
	Tetraedron trilobulatum			+		+	+	+	+
	Tetraedron regulare	+	+		+		+	+	
	Chlorella vulgaris	+	+	+	+	+	+	+	+
	Chlorococcum sp.		+		+		+	+	
	Nephrocytium agardhianum		+		+		+	+	+
	Oocystis naegelii	+	+	+	+	+	+	+	+
	Oocystis lacustris				+				

Ankistrodesmus falcatus	+	+					+	
Schroederia sp.							+	
Selenastrum sp.	+						+	
Closteriopsis sp.	+							+
Kirchneriella contorta		+	+	+	+	+	+	
Kirchneriella obesa				+		+		+
Pediastrum duplex								+
Crucigenia tetrapedia		+						
Quadrigula chodatii	+	+	+	+	+	+	+	+
Sphaerellopsis gelatinosa		+				+	+	+
Golenkinia paucispina	+	+	+	+	+	+	+	+
Lagerheimiella wratislaviensis					+	+		
Dictyosphaerium sp.					+	+	+	

Coelastrum microporum	+			+				+
Coelastrum reticulatum								+
Coelastrum morus	+	+	+			+	+	+
Westella botryoides		+						
Chlamydomonas simplex		+	+			+	+	
Palmella mucosa	+	+	+	+	+	+	+	+
Mougeotia parvula	+	+	+	+	+		+	
Cosmarium sp.							+	+
Cosmarium blyttii					+			
Euastrum ansatum				+	+	+		+
Cosmarium laeve	+	+	+	+	+	+	+	+
Spondylosium planum	+		+	+	+			

	Cosmarium contractum var. *rotundatum*					+	+	+	+
	Staurastrum sp.	+	+	+		+		+	+
	Staurodesmus triangularis	+	+	+	+	+		+	+
	Closterium venus	+	+		+	+			+
	Closterium gracile				+				
Cryptophyta	*Cryptomonas ovata*	+	+				+	+	+
	Cryptomonas erosa						+	+	
Dinophyta	*Peridiniopsis* sp.								+
	Gymnodinium sp.		+			+		+	
	Ceratium hirundinella	+						+	
Euglenophyta	*Trachelomonas volvocina*	+	+			+	+	+	+
	Euglena sp.	+	+	+	+	+	+	+	+

Bacillariophyta	*Cyclotella* sp.			+	+	+	+	+	+
	Achnanthes sp.	+	+	+	+	+	+	+	+
	Navicula sp.	+		+					
	Synedra acus	+	+	+	+	+		+	+
	Fragilaria sp.	+	+	+	+			+	+
	Gomphonema sp.						+		
	Cymbella sp.					+	+		+
	Nitzschia sp.	+	+			+	+		

Table S2. Relative biomass proportions of phytoplankton under different nutrient treatments.

Season	Relative biomass (%)	C	N	P	N+P
Winter	Cyanobacteriophyta	51.58	58.16	54.66	55.15
	Chlorophyta	44.99	37.22	41.27	39.19
	Cryptophyta	0.05	0.19	0.00	0.00
	Dinophyta	0.56	0.38	0.00	0.00
	Euglenophyta	1.07	3.03	0.84	0.34
	Bacillariophyta	1.75	1.02	3.24	5.32
Spring	Cyanobacteriophyta	14.03	5.67	6.22	3.25
	Chlorophyta	59.47	67.49	73.60	71.68
	Cryptophyta	0.00	0.86	0.37	0.21
	Dinophyta	0.43	0.00	0.54	3.51
	Euglenophyta	20.40	7.72	13.50	14.63
	Bacillariophyta	5.66	18.27	5.77	6.71
